# Consistency in identity-related sequential decisions

**DOI:** 10.1371/journal.pone.0260048

**Published:** 2021-12-08

**Authors:** Dikla Perez, Yael Steinhart, Amir Grinstein, Meike Morren

**Affiliations:** 1 The Graduate School of Business Administration, Bar-Ilan University, Ramat Gan, Israel; 2 Coller School of Management, Tel-Aviv University, Tel-Aviv, Israel; 3 D’Amore-McKim School of Business, Northeastern University, Boston, Massachusetts, United States of America; 4 Faculty of Economics and Business Administration, Vrije Universiteit Amsterdam, Amsterdam, the Netherlands; University of Haifa, ISRAEL

## Abstract

Consumers often make decisions that reflect either personal or social identities. In many cases, such decisions are made along a sequence. Our research introduces a central factor that influences consumers’ likelihood of expressing a consistent identity type along a sequence of decisions: the extent to which their usage of the product involved in the first decision is expected to be observable by others (the product’s *expected visibility*). A field experiment, and four lab studies, coupled with an internal meta-analysis, show that when the product involved in the first decision has high (as opposed to low) expected visibility, consumers are more likely to make a subsequent decision that is consistent with the first. Results show that self-presentation mediates this effect, and suggest that low integration between the identities involved in the decisions might attenuate it. Our findings offer implications for identity research and practical implications for marketers seeking to develop products and design communications that encourage consistent (or inconsistent) behavior.

## Introduction

Consumers often make decisions that relate to identities that they wish to express and communicate to others [1–4]. In many cases, the decision emphasizes an aspect of the consumer’s personal identity (e.g., purchasing a product that reflects a unique personal preference, such as a t-shirt in one’s favorite color), a social identity (e.g., purchasing a t-shirt of one’s favorite sports team) or both (purchasing a t-shirt of one’s favorite sports team in a unique favorite color) [1–3]. Moreover, it is common for such identity-related consumption decisions to be made along sequences, such as when shopping in a mall or running errands. Though many studies in marketing and psychology have examined the tendency to make consistent or inconsistent sequential decisions in a variety of contexts [5–10], very few have discussed the consistency of these decisions in the context of expressed identity. The current research sheds light on this issue by identifying a product characteristic that encourages consumers to consistently express one type of identity more prominently, either social or personal, along a sequence of decisions.

Specifically, we focus on the extent to which the consumer perceives that others will be able to observe him or her using or consuming the product involved in the first decision in a sequence—a characteristic we refer to as the product’s *expected visibility* [5, 11–13]. Examples of products that typically have high expected visibility include hats and t-shirts, whereas products with lower expected visibility include batteries or pajamas. Prior studies have shown that a product’s expected visibility can influence consumer behavior [12], such as propensity to generate positive word of mouth [11]. Herein, drawing from research on product ownership and usage as an identity signaling tool [2, 14, 15], we suggest that when a consumer is faced with a decision regarding a product with high rather than low expected visibility, he or she becomes more likely to express a consistent identity across subsequent identity-related decisions.

We distinguish the expected visibility of the consumed product from the visibility of the consumer when he or she is making a decision regarding that product—which we refer to as the *publicity* of the decision [16, 17]. An example of a high-publicity purchasing situation is shopping in a retail store; an example of a purchasing situation with low publicity is shopping online. We show that a product’s expected visibility influences consumers’ decision consistency regardless of the publicity of the decisions themselves.

We further propose that self-presentation concerns serve as an underlying mechanism for the effect observed. Specifically, we suggest that a consumer who is faced with a decision regarding a product with high expected visibility is likely to experience heightened awareness regarding the manner in which s/he presents oneself to others, and specifically, the identity that one expresses [16, 18–21]. These self-presentation concerns, in turn, may encourage the consumer to retain consistency, in terms of the identity s/he expresses, when making a subsequent decision. In contrast, when the product involved in the first decision is of low expected visibility (i.e., it is not expected to be seen by others when consumed), self-presentation concerns are not expected to become salient, and the consumer is no longer expected to show a heightened tendency for consistency in the product decision sequence.

Finally, we propose a moderator for the effect: the extent to which the consumer perceives the identities involved in the sequential decisions as being integrated, that is a perception of the linkage between an individual’s identities [22], and whether there is an integration or segregation between these identities [23]. Specifically, the effect of expected product visibility on decision consistency is more likely to be observed among consumers for whom the identities involved in the sequence of decisions are highly rather than poorly integrated.

In what follows, drawing from prior research on sequential decisions and visibility and identity theories, we lay the theoretical groundwork for our predictions regarding the effect of expected product visibility on consistency in sequential identity-related decisions, and regarding factors that may mediate and moderate this effect. We then describe five experiments in which we tested these predictions, measuring hypothetical choices, actual explicit and implicit product choices, and self-reported preferences, in the field, online, and in the lab. These experiments enable us to rule out a key alternative account: cognitive dissonance. Finally, we provide an initial indication as to whether a similar effect of product visibility might occur in other domains of sequential decisions.

This research has clear practical implications for marketers, as they suggest that presenting a product in a manner that highlights a certain level of expected visibility can influence consumers’ subsequent decisions. Notably, we provide a concrete example in which different images of an identical product—similar to images used in real-life marketing communications—are shown to influence consumers’ perceptions of a product’s expected visibility. Moreover, we show that the effect of expected product visibility on decision consistency is independent of the publicity of the decision itself. Accordingly, our observations are applicable both to scenarios in which consumers make purchase decisions in public (such as when shopping at retail stores) and to scenarios in which consumers purchase in private (such as when shopping online).

## 2. Theory development

### 2.1. Sequential decisions

A fundamental premise in the literature on sequential decisions is that, when individuals make multiple decisions, earlier decisions affect subsequent decisions. Several studies in this stream have focused on behavioral consistency, that is, whether a decision within a sequence of decisions is similar in nature to decisions made earlier in the sequence [7, 24–26]. These works consider a wide range of contexts and types of decision sequences, studying different products, such as decisions involving pens and CDs [6], soda and cereals [8], sporting events and beer [7], and chocolate cake and steaks [7]. Sequential decisions have also been examined in the context of non-consumption-related behaviors, such as donating to charity or volunteering in shelters [24, 25].

Studies examining individuals’ likelihood to make consistent as opposed to inconsistent sequential decisions have yielded mixed observations. On the one hand, some evidence shows that individuals tend to show consistent behavior when making sequential decisions [6, 7, 10, 24, 27, 28]. For example, if an individual consumes a tasty but unhealthy New York strip steak in a restaurant, s/he is then more likely to have a tasty but unhealthy chocolate cake at the same dinner [7]. Similarly, individuals who purchase a pro-social product (e.g., purchasing a picture when proceeds are partially donated to charity) subsequently become more likely to buy a second pro-social product (e.g., a present for someone) rather than to purchase a product for themselves [24]. Conversely, other studies have shown that individuals can demonstrate inconsistency in sequential decision processes [8, 15, 26–29]. In particular, a product or behavior chosen in a second decision may be different from or even opposite in nature to a product or behavior chosen in a previous decision [6, 8]. Drolet [8], for example, observes that participants show inconsistent preferences for private labels versus well-known brands. In that study, participants were asked to choose between a well-known brand of cereal and a private label of cereal. Then, in a second decision, they were asked to choose between a well-known brand of aspirin and a private-label brand of aspirin. Participants who had chosen a well-known brand in the first decision tended to select a private label in the second decision, and vice versa.

Our work addresses these mixed findings by identifying a factor that influences an individual’s tendency to make consistent as opposed to inconsistent sequential decisions (in terms of the identity expressed in the various decisions): the expected visibility of the product involved in the first decision.

### 2.2. Personal and social identities in sequential decision-making

Individuals possess multiple types of identities, many of which can be classified as personal or social identities. Each of these identities influences the individual’s attitudes and behavior [30–34], as well as his or her motivations in making decisions. In particular, social identity affects one’s motivation to assimilate with others and express a particular group affiliation [35, 36]. Personal identity, in turn, affects one’s motivation to affirm oneself as a unique individual, through actions and decisions that distinguish oneself from a social group [37]. Individuals can simultaneously express both types of identities or emphasize one as dominant over the other. For example, in purchase decisions, an individual can purchase a t-shirt that expresses a group affiliation, a t-shirt that expresses a unique personal preference, or a t-shirt that expresses both a group affiliation and a unique preference.

Existing theories dealing with personal and social identities and decision-making give rise to two predictions regarding whether engagement in a sequence of identity-related decisions should generate consistent identity expression. First, identity theory and the concept of identity salience [31, 33, 38] suggest that individuals are likely to show consistency when making sequential identity-related decisions. According to identity theory, the decision an individual makes at a given point in time is affected by the identity that is salient during the decision process [31, 33, 38, 39]. The salient identity might relate to characteristics such as one’s religious affiliation, workplace, place of residence, or political views. The identity that is salient depends on the situation and can be manipulated [30, 39]. This rationale suggests that decisions should be consistent if a specific identity is salient while an individual is making multiple decisions. That is, they should all express the same (salient) identity. Conversely, according to optimal distinctiveness theory [40], the individual has conflicting needs to express social- and self-identity. Brewer’s [40] theory suggests that individuals wish to reconcile these conflicting needs by avoiding excessive signaling of either personal or social identities and striving for balance between the two identities. Overall, optimal distinctiveness theory suggests that individuals are likely to show inconsistent behavior in sequential identity-related decisions. Following this rationale, when the first decision in a sequence expresses personal identity, the individual should make a second decision that expresses social identity, and vice versa. The current research reconciles the two conflicting predictions by proposing conditions under which consumers express consistent identities throughout a sequence of decisions.

### 2.3. Product visibility

Product visibility has been explored in the context of consumer behavior from two different perspectives. One stream of studies has examined the role of the visibility of the consumer at the time of making the purchase decision (i.e., the publicity of the decision) [16, 17]. In a purchasing situation involving high publicity (e.g., purchasing a product at a crowded store, or shouting out a bid in a traditional offline auction), the consumer is aware of the presence of others, and perceives that the decisions s/he makes may be observed and judged by others. The extent to which a consumer’s purchase behavior is visible has been shown to have a dramatic influence on consumers’ decisions. For example, Bateson and Roberts [14] drew a pair of eyes on an “honesty box” used to collect money for drinks in a university coffee room, creating an effect similar to someone watching individuals’ behavior. The authors observed that coffee room patrons put substantially larger amounts of money in that box than in an honesty box displaying a control image. Another study evaluated feelings of cognitive dissonance among participants who were asked to engage in behavior that was inconsistent with their attitudes. The authors observed that when a mirror was placed in the experimental environment—creating the feeling that “someone was watching”—participants experienced higher cognitive dissonance than they did when no mirror was present [17] (see section 3.2 for further discussion of cognitive dissonance).

Another stream of research focuses on the expected visibility of the consumed product, defined above [11–13], a construct that is distinct from the publicity of the purchase decision. The expected visibility of a consumed product has also been shown to influence consumers’ behavior. For example, Kulviwat and colleagues [12] observe that the relationship between social influence and adoption intention of an innovative product is stronger when the product expected visibility is high rather than it is low (consume in private). Herein, we focus on the effect of expected product visibility on the extent to which consumers make consistent sequential decisions. Notably, we predict that this effect will take regardless of the publicity of the purchase decision.

### 2.4 Self-presentation

We argue that the underlying mechanism that drives the effect of expected product visibility on consistency in identity-related sequential decisions relates to the construct of self-presentation. Self-presentation, which is also referred to as impression management [18–21], is generally defined as the process by which an individual tries to influence others’ impressions about her/himself. Indeed, individuals often seek to act in ways that will encourage others to adopt certain perceptions of them, particularly in light of the fact that perceptions can influence the manner in which one is treated [20, 41]. Schneider [42] specifies conditions that trigger engagement in self-presentation. Specifically, he suggests that self-presentation concerns are particularly likely to be elevated in situations where one’s image is relevant, such as in public situations [43]. Numerous studies lend support to this proposition [44–47], and specifically suggest that self-presentation concerns can trigger behaviors that are likely to maximize approval in social situations [48].

Building on these observations, we propose that the anticipation of being seen by others while consuming a product is likely to raise self-presentation concerns, i.e., to make consumers aware of how others perceive them (even if a decision regarding that product is made in private) [16, 18, 19, 21]. These concerns, in turn, are likely to elevate consumers’ propensity to portray themselves in a positive manner. We further suggest that consistency can be considered as a desirable attribute that one might seek to signal in order to convey a positive self-image [5, 49, 50]. For example, Aaker [5] shows that consumers find it important to maintain a consistent self-scheme and, accordingly, choose brands that help them in maintaining a consistent self-scheme presentation. Accordingly, consumers experiencing self-presentation concerns may become more likely to display consistent behavior. The latter proposition is supported by observations that self-presentation concerns, arising in public situations, can reduce the likelihood of displaying inconsistency between attitudes and behavior [19, 51, 52]. Taken together, these ideas suggest that the extent to which an individual is experiencing self-presentation concerns might mediate the effect of expected product visibility on consumers’ tendency to make consistent (as opposed to inconsistent) identity-related sequential decisions.

### 2.5. Integration between identities

Since each individual has different schemas, roles and identities, scholars [22, 23, 53] have explored how these identities can be integrated (and when they fail to be integrated) within an individual and have put forward propositions regarding the effect of such integration on behavior. Deaux [22], for example, has developed an integration model that consists of the linkage between individual’s identities. In the same vein, Aron, Aron, and Smolan [23] utilize a single-item scale to measure integration or segregation between identities. They present a model of self-concept that shows interconnectedness and connection using overlapping circles that represent the level of inclusion of other in the self (see S1 Fig).

Several empirical studies have provided evidence for the effects of identity-integration level on attitudes and behaviors. For example, Davis, Green and Reed, [54], use Aron and colleagues’ [23] overlapping circles approach to measure the level of inclusion of the natural environment in the self. They subsequently show that people who felt more connected to the environment were more likely to engage in pro-environmental behaviors compared with those who did not feel as connected. Another stream of studies focuses on identity integration among bicultural individuals [55–57]. Research in this area has shown that the extent to which individuals perceive their cultural identities as “compatible” (as opposed to “oppositional”) influences the extent to which they feel at harmony with their different identities.

Building on the works cited above, we argue that the integration level between the identities involved in a sequence of identity-related decisions can moderate the effect of expected product visibility on consistency of those decisions. Specifically, we argue that the effect is more likely to be significant among individuals for whom the corresponding identities are integrated rather than segregated. We predict that when the identity expressed in the first decision is highly connected to the identity expressed in the second decision, individuals are more likely to feel in harmony with this identity, and such harmony may generate a sense of congruency when making sequential decisions. Therefore, when experiencing this integration, individuals will be more prone to embrace the effect of expected product visibility in triggering consistency between decisions. However, when the identity expressed in the first decision is weakly connected to the identity expressed in the second decision, individuals are less likely to adopt a congruent mindset, and therefore the effect of expected product visibility on consistency will be attenuated.

## 3. The present research

### 3.1. Hypotheses

Summing up the ideas outlined in the previous section, we put forward the following hypotheses:

**H1:** In a set of sequential identity-related product decisions, an individual is more likely to engage in consistent behavior—i.e., to make a second decision that emphasizes the same (personal or social) identity as the first decision—when the product involved in the first decision is expected to be consumed in high-visibility rather than low-visibility circumstances.
**H2:** Heightened self-presentation concerns mediate the impact of the expected visibility of the consumed product on the likelihood of engaging in consistent behavior.
**H3:** Low integration between the different identities involved in the sequential decisions will diminish the impact of expected visibility of the consumed product on the likelihood of engaging in consistent behavior.

### 3.2. Plausible alternative account: Cognitive dissonance

In addressing our hypotheses, it is necessary to take into account cognitive dissonance as an alternative underlying mechanism, beyond self-presentation concerns elicited by expected product visibility, that might cause consumers to make consistent or inconsistent sequential decisions.

Consistency in individual behavior has been examined in psychological studies of cognitive dissonance while evaluating consistency between attitudes and behavior [21, 58–60]. This stream of research has shown that, given a behavior that does not align with an individual’s attitudes, the visibility of that behavior has a critical effect on the capacity of that behavior to elicit cognitive dissonance, as well as on the degree to which individuals are likely to engage in that behavior. Specifically, Tedeschi and colleagues [21] suggest that cognitive dissonance is more likely to generate behavior that is consistent with one’s attitudes when the behavior takes place in public rather than in private [19, 51, 52]. Notably, the cognitive dissonance literature has examined the role of the visibility (i.e., the publicity) of a *decision* in affecting consumers’ propensity to engage in behavior that is consistent with their attitudes. To our knowledge, studies in this domain have not examined how the expected visibility of a consumed product at the time of usage affects the propensity to make consistent sequential decisions, even in cases in which the decision itself is made in private (such as when purchasing a product online).

Cognitive dissonance inherently involves a conflict between one’s attitudes and behaviors [17, 61, 62], where the intensity of this conflict is expected to be elevated when the behavior takes place in public as opposed to private circumstances [19, 51, 52]. We argue that, in the context we explore, a product’s expected visibility can affect propensity to engage in consistent behavior even in the absence of conflict. That is, we examine consistency with regard to different aspects of one’s identity—which do not necessarily conflict with one another.

### 3.3 Outline of studies

We conducted four lab and online experiments, and a field experiment to test our hypotheses and to rule out an alternative explanation. Following prior work [6, 7, 8, 27, 28], the design of each of the five experiments consisted of presenting participants with two sequential choices. In our experiments we manipulated the expected visibility of the product involved in the first decision, and evaluating whether the identity type (social or personal) emphasized in the second choice matched the identity type emphasized in the first choice. Emphasizing the same identity in both choices represents consistency in identity-related sequential decisions. Experiments were conducted both in public and in private settings, enabling us to confirm that our results were robust across different levels of decision publicity.

The studies approved by the Tel-Aviv University Ethics Committee (11.24.2015). All participants but the field experiment participants signed an online written consent. We note that the data were analyzed anonymously.

## 4. Experiment 1—Exploring the effect of expected visibility on consistency on identity related sequential decisions

In this experiment we aimed to provide evidence regarding the effect of expected product visibility on consumers’ likelihood of engaging in consistent behavior when they are faced with two sequential choices in the lab.

We expect that in cases in which the expected visibility of the product’s usage environment can be manipulated (to convey either high or low expected visibility of the product) the effect of expected visibility on consistency is likely to occur. To examine this notion, we manipulated the visibility level of a service and presented participants with two identity-related decisions in a sequence.

Furthermore, in this experiment we sought to rule out cognitive dissonance as underlying mechanisms for the effect of expected product visibility on decision consistency. First, we explicitly aimed to show that the effect occurs even when the decision itself is not public (in an online experiment). We executed the study in a low-publicity setting, thereby minimizing the likelihood that participants would experience cognitive dissonance due to the publicity of the decision.

### 4.1 Method

#### 4.1.1 Participants and design

One hundred seventy-nine panel participants (*M*_age_ = 29.22, *SD*_age_ = 4.45, 64.8% female) were paid to take part in an online study. All participants were residents of major cities, where they had lived for an average period of 16.5 years (*SD* = 11.66). Participants were each randomly assigned to one of two expected-visibility conditions (high vs. low) in a between-subjects design.

#### 4.1.2 Procedure and measures

Participants were presented with an opportunity to participate in a new municipal service for which only city residents are eligible. This service involves participating in “city history meetings” with professional tour guides, who share notes about the history of the city and its uniqueness. Participants were told that registration for the service is free, and that the purpose of the meetings is to increase residents’ levels of identification with the city. Participation in the meetings was described as either high or low in expected visibility. Accordingly, we asked all participants to make a decision (first decision in the sequence). Specifically, participants in the high-expected-visibility condition were told that the meetings would take place in a group setting and that they could choose one of the following two types of locations for the meeting: (a) one of the city’s community centers or (b) the living room of one of the participants’ homes. 90% of participants chose a place in one of the city’s community centers as their preferred location for the meeting. Participants in the low-expected-visibility condition were told that the meetings would take place in a one-on-one setting, and they could choose one of the following types of locations: (a) a private office in one of the city’s community centers or (b) in the participant’s home. 60.8% of participants chose an office in one of the city’s community centers as their preferred location for the meeting. Then, all participants were asked to assume that they were about to be invited to a “summer nights” event (a festival featuring artists’ performances and fairs) in a city park and that the organizers were considering giving free t-shirts to attendees. The organizers are debating between two t-shirt options and are asking the participant, as a potential attendee, which t-shirt he or she prefers: a t-shirt in the participant’s favorite color, such that each participant will have a t-shirt reflecting his or her own preferences, or a t-shirt featuring the logo of the city (an identical image for all attendees). Under these conditions, choosing the latter t-shirt—i.e., a t-shirt featuring the logo of the city—reflects behavior that is consistent with the first choice. Next, as elaborated in what follows, participants responded to several additional questions reflecting measures of interest, followed by a demographic questionnaire.

#### 4.1.3 Cognitive dissonance

Participants were presented with a set of nine questions with regard to cognitive dissonance [63]. Specifically, participants were asked to indicate their feelings with regard to their first decision (the location of the city history meetings). For example, participants were asked to indicate on a 7-point scale (1 = not at all to 7 = very much) “To what extent do you feel regret about your decision?” or “To what extent do you feel disappointment from your decision?” These items were highly correlated (α = .919), and we averaged these ratings into a single measure of cognitive dissonance.

#### 4.1.4 Expected-visibility manipulation check

Participants were asked to rate their level of agreement with the following statements on a 7-point scale: “Many people are expected to know that I took part in the city history meetings”, “Friends are expected to know that I took part in the city history meetings”, and “Other people are expected to know that took part in the city history meetings” These items were highly correlated (α = .881) and were averaged into a single measure of expected visibility.

### 4.2 Results and discussion

#### 4.2.1 Expected-visibility manipulation check

As expected, participants in the high-expected-visibility condition assigned higher ratings to the expected visibility of the service (city history meetings in a group setting; *M* = 3.28, *SD* = 1.56) than did participants in the low-expected-visibility condition (city history meetings in a one-on-one setting; *M* = 2.68, *SD* = 1.79, *t*_(177)_ = 2.366, *p* < .019).

#### 4.2.2 Consistency of hypothetical choices

We conducted a logistic regression in which hypothetical t-shirt choice (0 = a t-shirt in the participant’s favorite color; 1 = a t-shirt featuring the city logo) was the dependent variable, and the expected-visibility condition (0 = low expected visibility; 1 = high-expected visibility) was the predictor. In line with H1, the effect of expected-visibility condition on participants’ choices was significant (Wald_(1)_ = 4.51, *p =* .033). Participants in the high-expected-visibility condition were more likely than participants in the low-expected visibility condition to choose the t-shirt featuring the logo of the city (reflecting participants’ social identity as residents of that city). Specifically, 54.0% of participants in the high-expected-visibility condition selected the t-shirt reflecting consistent behavior, as compared with 38.0% of participants in the low-expected-visibility condition (χ^2^(1) = 4.551, *p* = .033).

#### 4.2.3 Ruling out cognitive dissonance as an alternative explanation

A *t*-test analysis revealed no significant differences in cognitive dissonance between the high-expected-visibility condition (*M* = 1.70, *SD* = 1.04) and the low-expected-visibility condition (*M* = 1.72, *SD* = .93, *t*_(177)_ = .107, *p* >.1).

## 5. Experiment 2—Exploring the underlying mechanism: Self-presentation concerns

In this experiment we aimed to achieve two goals. First, we aimed to replicate the effect of expected product visibility on consistency in sequential decisions, and to extend our conclusions to circumstances in which the product involved in the first decision emphasizes one’s personal identity rather than one’s social identity (the identity emphasized in Experiment 1). Second, we sought to investigate whether heightened self-presentation concerns [18, 19] mediate the effect (H2).

### 5.1. Method

#### 5.1.1 Participants and design

Seventy-eight student participants (*M*_*age*_ = 22.60, *SD*_*age*_ = 2.05, 61.5% women) took part in an online study in exchange for course credit. Participants were randomly assigned to two conditions in a between-subjects design. Each condition corresponded to the expected visibility level of the consumed product involved in the first decision in a sequence (expected visibility: high expected visibility or low expected visibility).

#### 5.1.2 Procedure and measures

Participants were presented with a description of a product—a t-shirt—and were told they might win the t-shirt as a prize for their participation in the study. The t-shirt was described as emphasizing the participant’s personal identity (i.e., a t-shirt in the participant’s favorite color), and as a first decision participants were asked to choose their favorite color out of the 5 colors that were available (Purple, White, Red, Blue, or Black) for the product they might win. The product description further indicated that the t-shirt was either high or low in expected visibility in accordance with each participant’s assigned expected product visibility condition. Participants in the low-expected-visibility condition were asked to assume that the t-shirt they would potentially win was one that they would wear at home, such that not many people would see it. Participants in the high-expected-visibility condition were asked to assume that they would wear the t-shirt when going out, such that many people might see it. 43.6% of participants chose a black shirt, 24.4% chose a blue shirt, 24.4% chose a purple shirt and 7.7% chose a white shirt.

Then, all participants were informed that they would also receive a pen as a token of appreciation for their participation. They were presented with two descriptions of two types of pens with corresponding pictures and were asked to choose the pen they would like to receive. The first pen was a silver pen with the school logo printed on it (emphasizing the participant’s social identity as a student at the school). The other pen was identical, but without the logo, and was described as being available in a variety of colors, so each participant could choose a pen according to his or her favorite color (emphasizing the participant’s personal identity). Under these conditions, choosing the latter pen—i.e., the pen emphasizing personal identity—reflects behavior that is consistent with the first choice.

#### 5.1.3 Expected-visibility manipulation check

Participants were asked to rate their level of agreement with two statements on a 7-point scale (1 = strongly disagree to 7 = strongly agree): “Other people are expected to know that I own the product without me telling them,” and “Other people are not expected to know that I own the product without me telling them.” We reversed participants’ ratings on the second item and averaged both items into a single measure (α = .73) to represent the expected visibility level of each t-shirt [13].

#### 5.1.4 Self-presentation concerns

Participants were asked to complete a self-presentation scale adapted from prior studies [64]. Specifically, participants rated their level of agreement with five items on a 7-point scale (1 = strongly disagree to 7 = strongly agree) such as “Choosing this t-shirt makes me more aware of myself,” and “Choosing this t-shirt increases concerns regarding my dressing style.”. The items were found to be highly correlated (α = .77), and we averaged them into a single measure of self-presentation concerns.

### 5.2. Results and discussion

#### 5.2.1 Expected-visibility manipulation check

As expected, participants in the high-expected-visibility condition assigned higher ratings to the expected visibility of their corresponding product (a t-shirt likely to be worn outside; *M =* 4.18, *SD =* 1.49) than did participants in the low-expected-visibility condition (a t-shirt likely to be worn at home; *M* = 3.05, *SD* = 1.51, *t*_(76)_ = 3.31, *p* < .001).

#### 5.2.2 Behavioral consistency in the second decision

We carried out a logistic regression in which pen choice (0 = a colored pen without a logo; 1 = a silver pen with a logo) was the dependent variable, and expected-visibility condition (0 = low expected visibility; 1 = high expected visibility) was the predictor. In line with H1, the effect of the expected-visibility condition was significant (Wald _(1)_ = 3.923, *p =* .048): Participants in the high-expected-visibility condition were more likely than participants in the low-expected-visibility condition to choose the pen representing consistent behavior (i.e., a pen in the participant’s favorite color). Specifically, 83.8% of participants in the high-expected-visibility condition selected the pen reflecting consistent behavior, as compared with 63.4% of participants in the low-expected-visibility condition (χ^2^(1) = 4.101, *p* = .043).

#### 5.2.3 Self-presentation concerns as the mediator

In line with our expectations, we found that the expected visibility level of the product involved in the first decision was positively associated with the extent to which participants expressed self-presentation concerns (*b* = .97, *SE* = .28, 95% CI: .4241 to 1.522). Bootstrapping analysis [65] confirmed that the effect of expected visibility (0 = low visibility 1 = high visibility) on the likelihood of engaging in consistent behavior (i.e., choosing a colored pen in the second decision) was mediated by the level of self-presentation concerns (*b* = .39, *SE* = .30, 95% CI: .0160 to 1.180).

The results of Experiment 2 lend further support to H1, extending the results of Experiment 1 to a context in which the product involved in the first decision is a tangible one rather than a service and reflects a personal (rather than a social) identity, and in which decisions are made in a low-publicity environment (i.e., an online platform). Moreover, they suggest that when the product involved in the first decision has high expected visibility, individuals’ self-presentation concerns are triggered and consequently enhance their likelihood of engaging in consistent behavior.

## 6. Experiment 3—The moderating role of integration between identities

This experiment had two main goals. First, it aimed to replicate and extend our previous results regarding H1 by using a different product category (a mouse pad) and a different expected-visibility manipulation. Specifically, we manipulated the product’s expected visibility by presenting participants with an image featuring the product in a high- or low-visibility environment; the image resembled an advertising image that might be used in a realistic marketing communication situation. Note that, in contrast to the manipulations used in previous studies, the manipulation did not imply that the product would be used for different purposes and did not explicitly refer to the likelihood that others would see the product. This experiment further aimed to explore the moderation effect of integration between identities, proposed in H3 [22, 23, 53].

As in Experiment 1, we targeted residents of a one of the major cities of a country and offered them the opportunity to participate in a set of sequential decisions. In this experiment, participants were residents of “Metropolis,” a major cosmopolitan city with more than 3 million inhabitants. Metropolis, more than any other city in the country where it is located, has a large variety of cultural venues, restaurants, and shopping areas. Because of these unique features, Metropolis inhabitants tend to consider residency in the city as being part of their social identity (a fact that we pre-tested, as elaborated below).

### 6.1 Method

#### 6.1.1 Participants and design

One hundred fifty-four panel participants (*M*_*age*_ = 29.14, *SD*_*age*_ = 3.91, 55.2% women) were paid to take part in an online study. All participants were residents of Metropolis and had lived there for an average period of 11.6 years (*SD* = 9.91). Participants were each randomly assigned to one of two expected-visibility conditions (high vs. low) in a between-subjects design.

#### 6.1.2 Procedure and measures

**Fig 1:**
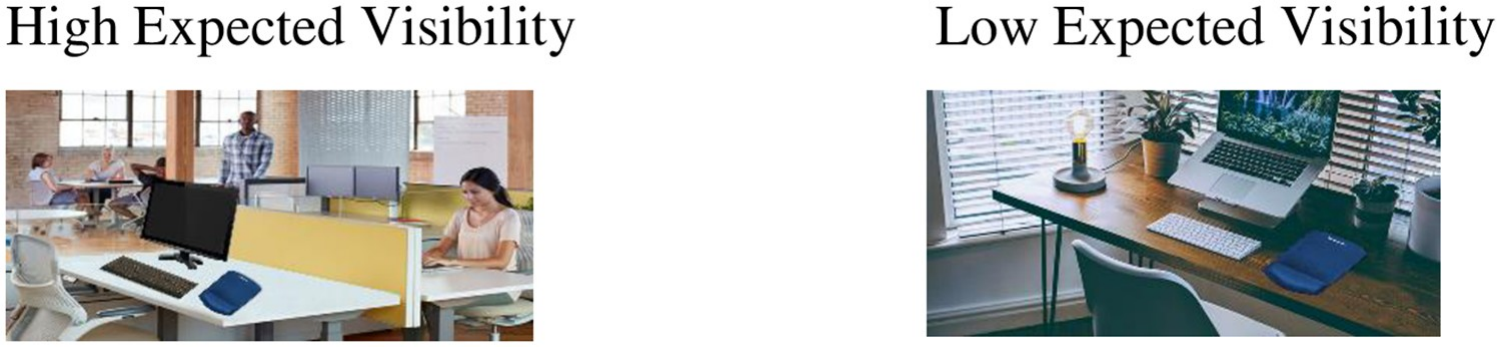
High- vs. low-expected visibility manipulation (Experiment 3).

We asked participants to assume that they had been given the opportunity to buy a product (a mouse pad) at an attractive price and that they could choose to print an image on the product. We then presented the participant with one of two pictures of the mouse pad, corresponding to his or her experimental condition (the expected-visibility manipulation). As shown in Fig 1, for participants in the high-expected-visibility condition, the picture featured the mouse pad in a high-visibility environment (an open space office in which people were present), whereas for participants in the low-expected-visibility condition the mouse pad was shown in a low-visibility environment (home office). As a first decision, the participant was then presented with two images and was asked which one he or she would like to print on the mouse pad. Both images implied that they emphasized the participant’s social identity as a resident of Metropolis and included a well-known slogan of Metropolis (we coded them image 1 or image 2, these codes were not presented to participants and the images were presented in a random order). No additional information with regard to the product’s expected visibility was provided. 70.8% chose image no. 2.

Then, all participants were asked to assume that they would receive a pen as a token of appreciation for their participation. They were asked to choose which type of sentence they would like to print on this pen: a sentence that reflects their unique personal identity or a sentence that reflects their identity as residents of Metropolis. Under these conditions, choosing the latter pen—i.e., a sentence that reflects the participant’s identity as a resident of Metropolis—constitutes behavior that is consistent with the first choice.

Next, as a measure for integration between identities, all participants were asked to indicate their level of agreement with the sentence: “I feel like I have two different identities: my personal identity and my identity as a resident of Metropolis” (1 = completely disagree to 7 = completely agree). Scores were reverse-coded to produce a measure of integration between identities, where a higher value indicated higher integration between a participant’s personal identity and his or her identity as a resident of Metropolis.

Finally, participants were asked to complete a set of manipulation check measures and a demographic questionnaire.

#### 6.1.3 Expected-visibility manipulation check

Participants were asked to rate their level of agreement with the following statements on a 7-point scale: “Many people are expected to know that I own this mouse pad”, “Friends are expected to know that I own this mouse pad without me telling them” and “Other people are expected to know that I own this mouse pad without me telling them.” These items were highly correlated (α = .85), and we averaged these ratings into a single measure of expected product visibility.

#### 6.1.4 Pre-test confirming Metropolis residency as a component of participants’ social identity

In a pre-test, we asked 84 panel participants (*M*_*age*_ = 30.9, *SD*_*age*_ = 4.07, 66.9% women), all residents of Metropolis and who had lived there for an average period of 12.3 years (*SD* = 11.05), to rate four items related to their social identity as residents of Metropolis on a 7-point scale (1 = not at all to 7 = very much): “Please rate the extent of your belongingness to this city,” “Please rate the extent to which you feel part of this city,” “Please rate the extent to which you feel that your identity as a resident of the city is part of your identity,” and “In your opinion, to what extent do you think it is important to preserve the identity of this city for its residents?” These four items were highly correlated (α = .95), and we averaged them into a single identity measure. According to a *t*-test, the extent to which participants rated their residency in Metropolis as a component of their social identity was significantly higher than the mid-scale (*M =* 4.83, *SD =* 1.65, *t*_(82)_ = 4.58, *p* < .001).

### 6.2 Results and discussion

#### 6.2.1 Expected-visibility manipulation check

As expected, participants in the high-expected-visibility condition (who were shown an image of the mouse pad in an open space office) assigned higher ratings to the expected visibility of the product (*M* = 3.67, *SD* = 1.59) than did participants in the low-expected-visibility condition (the mouse pad that was placed in a home office; *M* = 3.10, *SD* = 1.67, *t*_(152)_ = 2.16, *p* < .032).

#### 6.2.2 Main analysis

We conducted a logistic regression in which the dependent variable was participants’ choice of the sentence to be printed on the pen they would receive (0 = a sentence that reflects one’s unique personal identity; 1 = a sentence that reflects one’s identity as a resident of Metropolis), the predictor was the expected- visibility condition (0 = low-expected visibility; 1 = high-expected visibility); and integration between identities score served as the moderator (Process, Model 1), [65],. As shown in Table 1, in line with H1, expected-visibility condition had a significant effect on participants’ choices (*b* = 1.18, *SE* = 0.60, z(1) = 1.99, *p* = .047), such that being in the high-expected-visibility increased the likelihood of making a consistent choice (i.e., selecting a sentence that reflects one’s identity as a resident of Metropolis). Furthermore, we observed a marginally significant interaction between product visibility and integration between identities (*b* = 0.57, *SE* = 0.33, z (1) = 1.76, *p* = .08).

**Table 1:** The effect of expected visibility conditions (High vs. Low) and integration between identities on consistent behavior (Experiment 4).

	Identity expression in the second decision
Intercept	-2.06** (0.30)
Product visibility	1.18** (0.60)
Identity integration	-0.15 (0.16)
Product visibility× identity integration belongingness	0.57* (0.33)
Coxsnell	0.466*

Notes: In the regression reported in the table, the change in tendency toward consistency (reflected via identity expression in the second decision) is the dependent variable. As for the predictors, integration between identities is a continuous variable that takes values from 1 to 7 and is mean-centered; product visibility is a dummy variable (0 –low visibility, 1 –high visibility). Entries in the table represent unstandardized coefficients. Standard errors are reported in parentheses.

* *p* < .1,

** *p* < .05.

**Fig 2:**
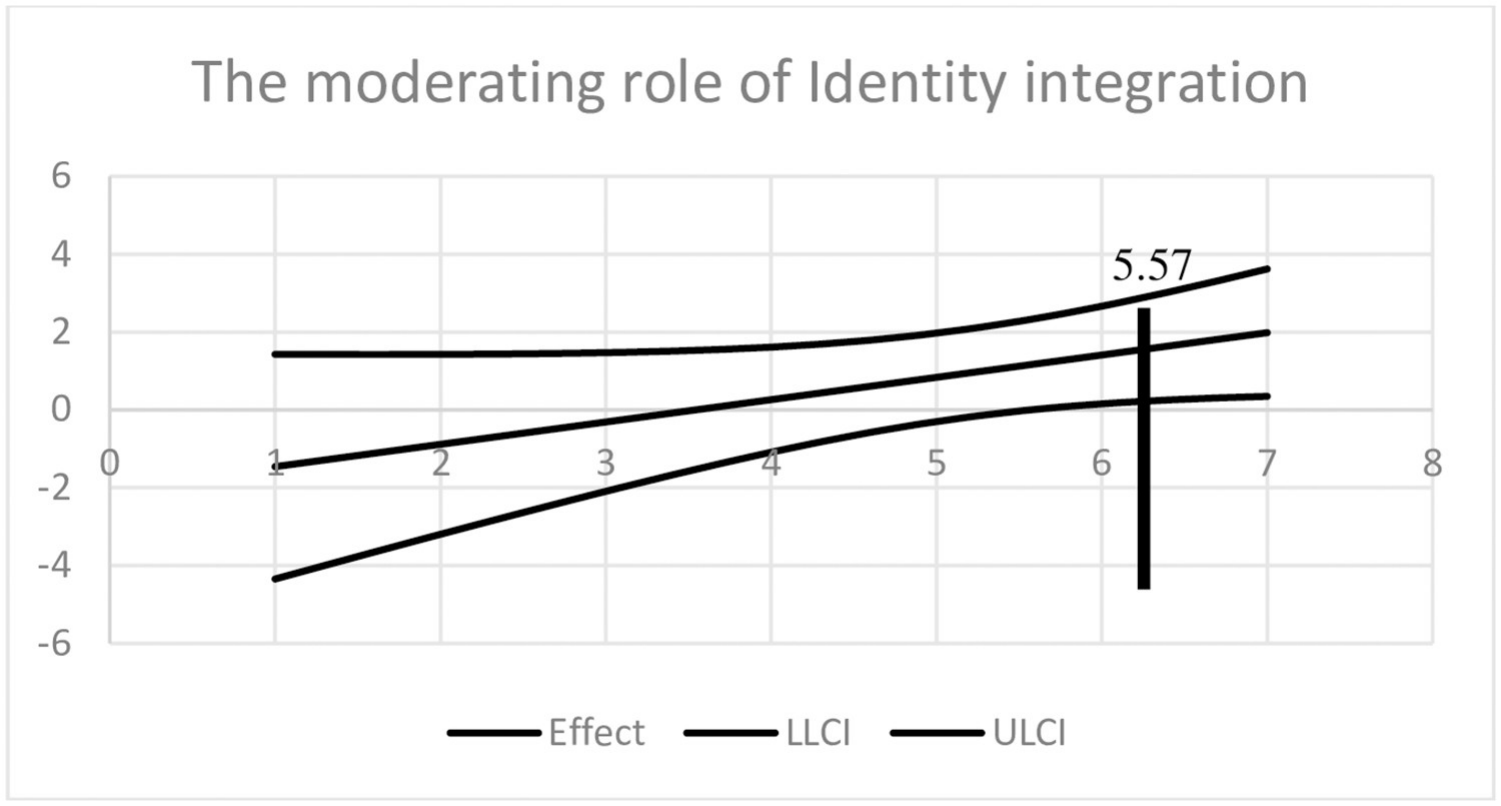
Floodlight analysis: The moderating role of identity integration (Experiment 3). Notes: The graph was drawn on the basis of a "floodlight" analysis (Disatnik and Steinhart 2015; Spiller, Fitzsimons, Lynch Jr, and McClelland 2013) of the effect of identity integration on tendency toward consistent behavior. Confidence bands are also presented, and the Johnson-Neyman points are obtained at *identity integration* = 5.57 (*p* = .05).

Because the integration between identities measure was a continuous variable, to gain further understanding of the interaction, we used the Johnson-Neyman “floodlight” approach that is recommended by Spiller et al. [66, 67]. As shown in Fig 2, the effect of expected product visibility on tendency toward consistent behavior in the second decision was significant only among participants with integration between identities levels exceeding 5.67 (61.69% of the participants; *B*_*JN*_ = 1.16, *SE* = 0.59, *p* = .05; see also S1 Table).

This experiment lends further support to H1. Moreover, the results lend support to the moderating role of integration between identities in the effect of expected product visibility on the tendency to make consistent identity-related decisions (H3). We further note that, as in Experiment 2, participants made their choices in a low-publicity online environment, similar to an online shopping setting.

## 7. Evidence of the effect of expected visibility while exploring actual behavior

In the following two experiments, we aimed to provide evidence regarding the effect of expected product visibility on consumers’ likelihood of engaging in consistent behavior when they are faced with two actual sequential choices. Experiment 4 provides evidence for this effect on a field setting while experiment 5 provides evidence for consistency in identity related actual choices in a lab setting.

### 7.1 Experiment 4—Field evidence

In this experiment we approached residents of a specific city (referred to in what follows as “Smallville”) and offered them the opportunity to make two sequential decisions. Smallville is relatively small, with fewer than 30,000 inhabitants. It has a unique history and atmosphere, and residents consider living in Smallville to be a strong aspect of their social identity (a fact that we confirmed in a pre-test; see below).

#### 7.1.1 Method

***7*.*1*.*1*.*1*** *Participants and design*. Participants comprised 50 shoppers in a Smallville supermarket (34% women), who were randomly assigned to two conditions in a between-subjects design. The specific supermarket is a neighborhood store, not part of any large retail chain, and the vast majority of its customers are residents of Smallville. Each condition corresponded to the expected visibility level of a consumed product that would be involved in the first of two decisions (expected visibility of the consumed product: high expected visibility or low expected visibility).

*7*.*1*.*1*.*2 Procedure*. At the entrance to the supermarket, we displayed a sign offering shoppers the opportunity to buy a t-shirt for the attractive price of $2.50 (see S2 Fig). Thus, the first decision was whether or not to purchase the t-shirt. The t-shirt had the Smallville logo printed on it (i.e., it expressed shoppers’ social identity; see pre-test described below). The t-shirt was presented to shoppers either as a high-expected-visibility product or as a low-expected-visibility product, as detailed in the following subsection. Shoppers who purchased a t-shirt (from an experimenter standing next to the sign) were then told to choose a pen as a free gift. Shoppers chose between two types of pens: either a black pen printed with the Smallville logo (i.e., a pen expressing the customer’s social identity), or a pen in the color of the shopper’s choice that did not display the Smallville logo, but was otherwise identical to the first pen (predominantly expressing the customer’s personal identity; see S3 Fig). Selection of the first type of pen was considered to be a “consistent” decision (as it emphasized the same identity as the t-shirt, namely, social identity), whereas selection of the second type of pen was considered to be an “inconsistent” decision.

*7*.*1*.*1*.*3 Expected-visibility manipulation*. We manipulated the expected visibility level of the t-shirt by creating two different signs, one for each expected-visibility condition (see S2 Fig). We displayed one sign at a time, alternating between them every hour. The sign presented to participants in the high-expected-visibility condition stated that the product being offered was a “t-shirt”. The sign presented to participants in the low-expected visibility condition stated that the product was a “pajama top”. Note that the product offered to participants was the same in both conditions; the only difference was in the words used to describe the product. In a pre-test comprising 85 individuals (*M*_*age*_ = 31.4, *SD*_*age*_ = 5.45, 46.8% women), participants indeed rated the expected visibility of the product higher when it was advertised as a “t-shirt” (*M =* 4.23, *SD =* 1.70) than when it was advertised as a “pajama top” (*M* = 2.71, *SD* = 2.00, *t*_(83)_ = 3.78, *p* < .0001). We further note that the publicity of participants’ decisions was identical in both conditions; specifically, the experiment took place in a public environment (the entrance to a supermarket).

*7*.*1*.*1*.*4 Pre-test confirming Smallville residency as a component of inhabitants’ social identity*. We recruited individuals through a Smallville Facebook group and asked them to respond to a short questionnaire. Thirty-nine participants (*M*_*age*_ = 50.8, *SD*_*age*_ = 8.59, 64.1% women) volunteered to take part in the pre-test. All participants were current residents of Smallville, and had lived there for an average of 16 years (*SD* = 8.73). Participants were presented with three questions related to their social identity as residents of the city. Specifically, we asked participants to rate the following items on a 7 point-scale (1 = *not at all* to 7 = *very much*): “Please rate the extent of your belongingness to this city”; “Please rate the extent to which you feel part of this city”; and, “In your opinion, to what extent do you think it is important to preserve the identity of this city for its residents?” These three items were highly correlated (α = .88) and were averaged into a single social-identity measure. Participants’ social-identity ratings (*M =* 4.64, *SD =* 1.46) were significantly higher than the mid-point of the scale (*M =* 4, *t*_(38)_ = 2.746, *p* < .01), indicating that they considered living in Smallville to be a meaningful component of their social identity.

#### 7.1.2. Results and discussion

In line with H1, 74% of participants in the high-expected-visibility condition selected the pen representing consistent behavior (i.e., the pen with the Smallville logo), compared with only 35.5% of participants in the low-expected-visibility condition. A logistic regression of participants’ pen choices (0 = a colored pen; 1 = a pen with the Smallville logo) as a function of expected-visibility condition (0 = low expected visibility; 1 = high expected visibility) revealed a marginally significant effect (Wald_(1)_ = 3.52, *p* = .06). These results, obtained from consumers making real choices, provide evidence in support of H1 from the field.

As noted above, participants in this experiment made choices under high-publicity circumstances, i.e., in a public place where others could see them. These circumstances may have affected participants’ likelihood of engaging in consistent behavior. We addressed this concern in our subsequent experiments by presenting participants with the opportunity to make decisions in private, where only a few people if any, could see their choices.

### 7.2 Experiment 5—Actual behavior in a lab setting

Experiment 5 aimed to extend the results of prior experiments by showing that the effect of expected visibility on decision consistency takes place even when the consumer is not explicitly aware that he or she is making a decision (i.e., the decision is implicit, or non-intentional). We further sought to extend our prior experiments by considering a scenario in which some participants were faced with a first decision relating to personal identity whereas others were faced with a first decision relating to social identity. This enabled us to measure the fit between the identity reflected in the first decision and the identity expressed in a subsequent product decision.

#### 7.2.1 Method

*7*.*2*.*1*.*1 Participants and design*. One hundred-two student participants (*M*_*age*_ = 22.57, *SD*_*age*_ = 2.17, 53.9% women) were invited to the laboratory and took part in both an online and offline study in exchange for course credit. Participants were randomly assigned to four conditions in a 2 × 2 between-subjects design: identity emphasized by the product involved in the first decision (personal identity or social identity) × expected visibility of the product involved in the first decision (high expected visibility or low expected visibility).

*7*.*2*.*1*.*2 Procedure and measures*. Participants were presented with a description of a product (a t-shirt) and were told they might win the t-shirt as a prize for their participation in the study. The product descriptions corresponded to participants’ assigned conditions. Specifically, each participant in the personal-identity condition was told that the t-shirt would be in his or her favorite color, whereas participants in the social-identity condition were told that the t-shirt was printed with the logo of the school they attended. Moreover, the t-shirt was described as either high or low in expected visibility: Participants in the low-expected-visibility condition were asked to imagine that the t-shirt was one that they would be likely to wear at home, such that not many people would see it. Participants in the high-expected-visibility condition were asked to imagine that they would wear the t-shirt when going out, such that many people might see it.

Then, all participants were given the opportunity to select a pen from two sets of pens, each of which expressed a different type of identity. Specifically, on the desk next to each participant, we had placed two pen holders, one containing silver pens with the school logo printed on them (reflecting participants’ social identities), and the other containing identical pens without the logo. The latter pens were in a variety of colors, so each participant could choose a pen according to his or her favorite color (thereby emphasizing personal identity). All participants were asked to fill out a participation form provided by the researcher using a pen of their choice. They were also informed that they could keep the pen they had chosen as a token of appreciation for their participation. We emphasize that this selection was not an explicit choice–meaning that participants were not explicitly told that they must choose a pen.

After filling out the form, participants proceeded to complete an online survey and to provide demographic information while the researcher collected the participation forms and discreetly marked participants’ pen choices. We subsequently determined whether each participant’s choice reflected consistent or inconsistent behavior. For participants in the social-identity condition, choosing a pen with the school logo reflected consistent behavior. For participants in the personal-identity condition, choosing a pen in a color of the participant’s choice reflected consistent behavior.

*7*.*2*.*1*.*3 Expected-visibility manipulation check*. Participants were asked to use a 7-point scale (1 = strongly disagree to 7 = strongly agree) to rate their level of agreement with the statement: “Other people are not expected to know that I own the product without me telling them.” We reversed participants’ ratings of this statement to represent the expected visibility level of each t-shirt [13].

*7*.*2*.*1*.*4 Emphasized identity manipulation check*. As a manipulation check, participants were asked to rate the extent to which they perceived the t-shirts that had been described to them as emphasizing a social identity [68]. Specifically, they were asked to use a 7-point scale rate their level of agreement (1 = strongly disagree to 7 = strongly agree) with the statement: “The presented product reflects the social identity of its user.”

#### 7.2.2 Results and discussion

*7*.*2*.*2*.*1 Expected-visibility manipulation check*. Participants in the high-expected-visibility condition indicated that they perceived their respective products as being higher in expected visibility (*M =* 4.40, *SD =* 1.89) than did participants in the low-expected-visibility condition (*M* = 3.76, *SD* = 1.79, *F*(1, 98) = 3.39, *p* = .07); this difference was marginally significant. The emphasized-identity condition had no effect on product visibility ratings (*p* > .4).

*7*.*2*.*2*.*2 Emphasized identity manipulation check*. As expected, participants in the social-identity condition perceived their respective products as reflecting one’s social identity to a greater extent (*M =* 4.58, *SD =* 1.56) than did participants in the personal-identity condition (*M* = 3.26, *SD* = 1.79, *F*(1, 98) = 16.55, *p* < .001). Expected-visibility condition had no effect on participants’ perceptions regarding the identity type that was emphasized (*p* > .2).

*7*.*2*.*2*.*3 Consistency of actual choices*. In line with H1, a logistic regression on participants’ actual choices (0 = inconsistent behavior; 1 = consistent behavior) revealed a significant effect of the expected-visibility on behavioral consistency in the second decision (Wald_(1)_ = 4.73, *p =* .030): Participants in the high-expected-visibility condition were more likely than participants in the low-visibility condition to choose the pen representing consistent behavior (i.e., a logo pen for the social-identity condition and a colored pen for the personal-identity condition). Specifically, 57.7% of participants in the high-expected-visibility condition selected the pen reflecting consistent behavior, as compared with 36.0% of participants in the low-expected-visibility condition (χ^2^(1) = 4.81, *p =* .028). Among participants in the social-identity condition, 60.9% of participants in the high-expected visibility condition selected the logo pen, and among participants in the personal-identity condition, 55.2% of participants in the high-visibility condition selected the colored pen.

Taken together, the results of Experiments 4 and 5 replicate and extend the results of Experiments 1 to 3 as elaborated above also for actual identity related sequential decisions.

## 8. Converging evidence through internal meta-analysis

We conducted a small-scale internal meta-analysis based on 563 participants of Experiments 1–5. The goal of the internal meta-analysis was to provide converging evidence across all studies about the role of expected visibility in triggering decision consistency in a sequence of two decisions. This approach is in line with recent attempts to use quantitative methodology to strengthen the validity of behavioral research that is based on small samples [69, 70].

The predictor in the internal meta-analysis was the expected-visibility level of the product involved in the first decision in the sequence (i.e., high expected visibility or low expected visibility). The dependent variable was the extent to which participants’ second decisions were consistent with their first decisions.

We conducted the internal meta-analysis using the procedures described by Hunter and Schmidt [71] and recently applied by Tuk, Zhang and Sweldens [70] and by Perez, Stockheim, Tevet and Rubin [69]. In our experiments, we observed the effect of the expected visibility of the product involved in the first decision on participants’ displays of consistent behavior in sequential decisions. To synthesize the effect sizes, we recalculated all the effects in the studies as correlation coefficients [72]. We used these correlation coefficients to compute the weighted effect (corrected for sample size) of high/low visibility condition on likelihood of engaging in consistent behavior for each type of emphasized identity (see Table 2). Next, we calculated the standard error of the weighted effect size [73] and a 95% confidence interval around each weighted effect size [74]. A confidence interval that includes 0 suggests that the correlation obtained in the meta-analysis may not be different from 0 in the population of studies (i.e., the correlation is not significant).

**Table 2:** Experiments included in the internal meta-analysis.

Experiment	N	Statistics*	Value	r**	LCI	UCI
Experiment 1	179	Chi	4.55	.16	.15	.17
Experiment 2	78	Chi	4.10	.23	.21	.25
Experiment 3	154	Chi	3.39	.15	.14	.16
Experiment 4	50	Chi	3.63	.27	.24	.30
Experiment 5—social	52	Chi	2.70	.23	.19	.26
Experiment 5—personal	50	Chi	2.34	.22	.18	.25
Total	563			.187	.17	.20

* Chi-square tests were run on the 2×2 table of expected visibility of the consumed product (low versus high) with consistency (consistent choices versus inconsistent choices). The *t*-test examined the mean difference on a preference rating between two groups subjected to manipulations of expected visibility of the consumed product involved in the first decision (low versus high).

** Sample-size-weighted average correlation between expected visibility of the consumed product involved in the first decision and choices displaying consistent behavior.

**Fig 3:**
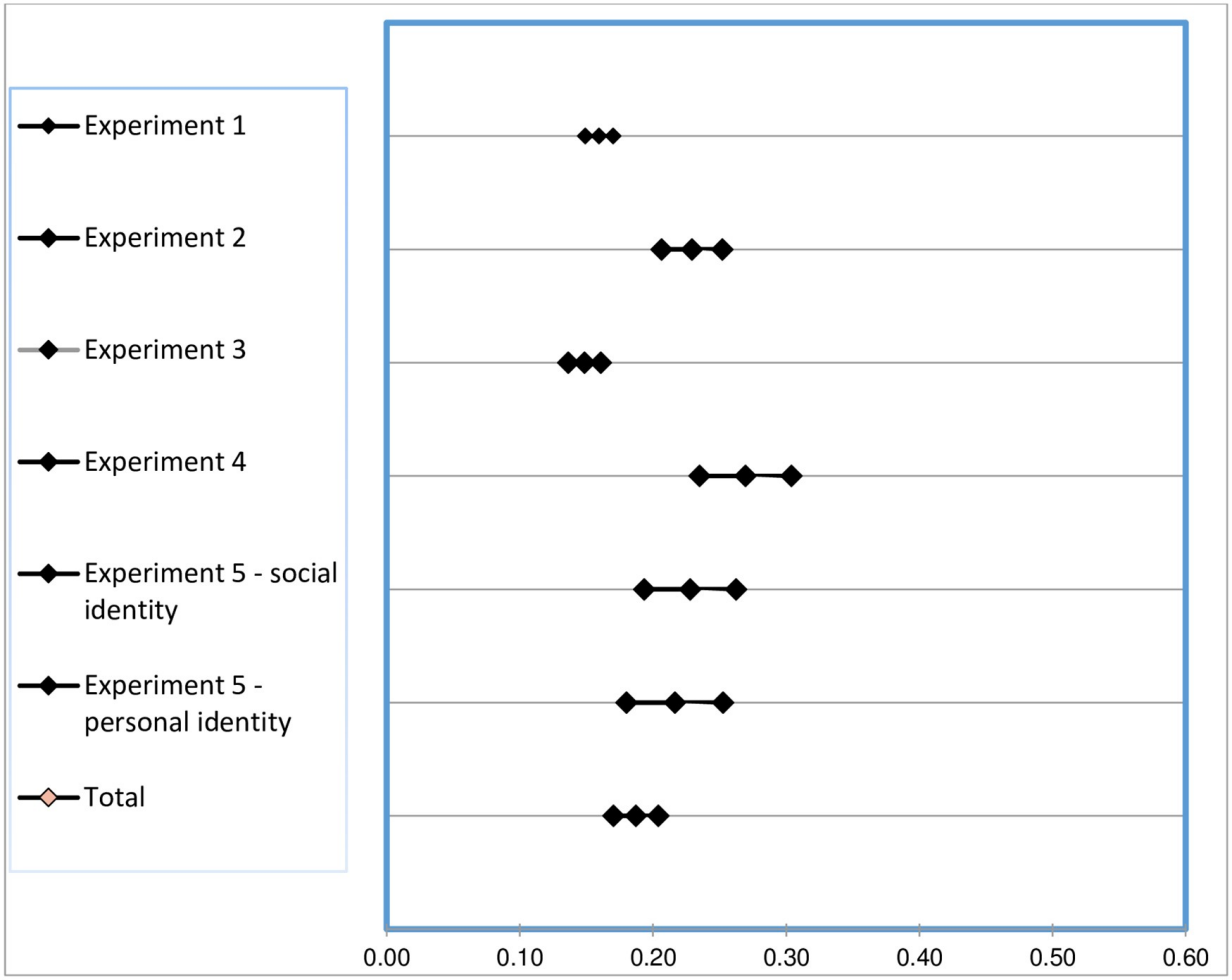
Effect sizes of the internal meta-analysis.

The results of the internal meta-analysis confirmed our expectations: The average weighted correlation coefficient across all studies had a value of .187 and the confidence intervals did not include 0 (95% CI: .171 to .204), indicating that, in our studies product visibility in the first decision was positively associated with participants’ likelihood of engaging in consistent behavior (see Fig 3).

## 9. Additional evidence regarding the role of expected visibility in decision consistency: Content analysis

Given that quite a few studies have considered sequential decisions in diverse domains [6, 8, 24, 28], we sought to explore whether our predictions regarding the role of expected visibility in decision consistency are potentially applicable to contexts other than identity expression. To this end, we analyzed 21 related studies from seven different research projects, which were conducted in different decision contexts. The selection of these studies was based on a systematic literature review of the marketing literature from 1966 to 2012. In this analysis, we identified products and non-consumption-related behaviors used to examine consistency or inconsistency in sequential product-related decisions. We relied on the studies’ conclusions, for each case, to determine whether the behaviors observed were consistent or inconsistent. Using two independent judges who were blind to the purpose of the research, we further classified the products on the basis of their expected visibility levels. Specifically, we relied on a content analysis procedure [75, 76] and asked two academic experts in the fields of marketing and psychology to rate, on a 7-point scale, the extent to which others are expected to see an individual consuming each product or service [11, 13] (see Table 3).

**Table 3:** Review of previous research: Products/behaviors used to examine consistency in sequential decisions.

Research article	Products/behaviors involved in a first decision	Products/behaviors involved in a second decision	Type of behavior	Average rating of the expected visibility of the product involved in the first decision across two expert judges (1 = low visibility, 7 = high visibility)	Average rating of the expected visibility of the product involved in the second decision across two expert judges (1 = low visibility, 7 = high visibility)
*Inherent rule of variability*	Well-known brand or private label CD player	Well-known brand or private label microwave	Inconsistent	2.00	2.00
Drolet (2002)	Well-known brand or private label color TV	Well-known brand or private label CD player	Inconsistent	2.00	2.00
	Well-known brand or private label batteries	Well-known brand or private label toaster	Inconsistent	1.50	1.50
	Well-known brand or private label toaster	Well-known brand or private label microwave	Inconsistent	1.50	2.00
	Well-known brand or private label cereal	Well-known brand or private label aspirin	Inconsistent	2.00	1.50
	Well-known brand or private label swabs	Well-known brand or private label aspirin	Inconsistent	2.00	1.50
	Well-known brand or private label soda	Well-known brand or private label aspirin	Inconsistent	2.00	1.50
	Well-known brand or private label sugar	Well-known brand or private label aspirin	Inconsistent	1.50	1.50
*Licensing*	Spend three hours a week teaching children in a homeless shelter	Purchase designer jeans	Inconsistent	3.50	6.50
Khan and Dhar (2006) [77]					
	Donate $100 to charity (online)	Purchase sunglasses	Inconsistent	1.50	7.00
*Goal accessibility*	Wear a hat on a bright sunny afternoon	Use sunscreen	Consistent	6.50	2.00
Fishbach, Dhar, and Zhang (2006)	Have a light lunch	Have a light dinner	Consistent	6.50	6.50
	Work out in a gym	Eat healthily	Consistent	7.00	2.00
*Shopping momentum*	Light bulb	Key chain	Consistent	1.50	2.00
Dhar, Huber, and Khan (2007)	Pen	Key chain	Consistent	5.50	2.00
	Car	Key chain	Consistent	7.00	2.00
*Costly signaling*	Purchase a photo under charitable-giving promotion	Purchase merchandise as a gift for someone else	Consistent	2.00	2.00
Gneezy et al. (2012)					
*Peak experience*	Purchase a high-price ticket for a baseball game	Purchase a high-price beer at a baseball game	Consistent	4.50	6.00
Dhar and Simonson (1999)					
	Dinner at a nice restaurant. Eat tasty but unhealthy New York steak	Eat a tasty but unhealthy chocolate cake	Consistent	5.50	5.50
*Foot in the door*	Answer a few questions for a survey	Allow five-six men to come into one’s house to classify the household’s products for two hours as part of a survey	Consistent	1.50	4.00
Freedman and Fraser (1966)					
	Put a small sign in the window to make citizens more aware of the need to drive carefully	Put a large sign in the front yard to make citizens more aware of the need to drive carefully	Consistent	7.00	7.00

The two judges independently rated the consumed products’ visibility and agreed on 95.23% of the 21 products. Disagreement between judges was resolved by discussion. Based on the judges’ ratings, we classified the products into two categories: low- and high expected visibility. For example, a ticket for a sporting event was classified as a highly visible product. In a study by Dhar and Simonson [7], purchasing such a product was associated with consistent behavior, such that individuals who purchased a high- or low-priced ticket also purchased high- or low-priced beer at the event, respectively. Conversely, cereal was classified as a low-expected-visibility product. In a study by Drolet [8], purchasing such a product was associated with inconsistent behavior, such that individuals who chose to purchase cereal from a private label (as opposed to a well-known brand) were likely to subsequently choose a second product of an opposite nature, i.e., from a well-known brand (as opposed to a private label), and vice versa.

Results indicated that the product involved in the first decision was perceived as high in expected visibility (rated above the mid-point of the expected-visibility scale) in 70% of studies in which consistent behavior was observed. Furthermore, in 100% of studies in which inconsistent behavior was observed, the product involved in the first decision was perceived as low in expected visibility (rated below the mid-point of the visibility scale). The two groups of studies did not differ in terms of the expected visibility ratings of the products involved in the second decision. Overall, this analysis supports the premise of H1 and highlights expected product visibility as an important factor in determining whether, in general, individuals’ product-related decisions will be consistent or inconsistent.

Moreover, to rule out the possibility that decision consistency in different studies was an artifact of the expected visibility of the product involved in the second decision, we analyzed judges’ ratings for the expected visibility of the product involved in the second decision. We did not find evidence indicating that the expected visibility of the product involved in the second decision drove consistency. Specifically, judges rated the product involved in the second decision as being high in expected visibility (i.e., rated above the mid-point on the expected visibility scale) in only 36.4% of studies in which consistent behavior was observed.

## 10. General discussion

The current research shows that, in a sequence of decisions regarding products that emphasize participants’ social or personal identities, the expected visibility of the product involved in the first decision has a role in determining whether or not the consumer will make consistent decisions throughout the sequence (i.e., will make choices that consistently emphasize the same type of identity, social or personal). Specifically, results of five experiments show that when a high-expected visibility product reflecting a given identity is involved in the first decision, participants tend to emphasize that same identity in the next decision in the sequence. Conversely, the results demonstrate an attenuation of this tendency when the product involved in the first decision is of low expected visibility. Our findings also suggest that self-presentation concerns underlie this pattern of behavior. Specifically, an initial decision that involves a high-expected-visibility product appears to activate a self-presentation process, guided by situational cues of social appropriateness. This tendency manifests in a need to appear consistent by emphasizing an identity identical to that emphasized in the first decision. Finally, findings show that the effect of expected product visibility on the tendency to engage in consistent behavior is attenuated when the integration between identities involved in the sequence is low.

Across the experiments, we invoked different social identities (identity as a student, residence in a city), exposed participants to different products (t-shirts, mouse pad, a municipal service), and different manipulations of expected visibility levels, and we measured different dependent variables (hypothetical choices, actual choices, explicit and implicit choices). In addition, we demonstrate our findings in the field, online, and in the lab. All findings support our main hypothesis (H1). Importantly, our findings suggest that the effect takes place regardless of the publicity of the decision itself (see S2 Table. for a summary of the results). Furthermore, we have ruled out cognitive dissonance, as alternative explanations.

### 10.1 Implications for identity research

The current research makes several contributions to the literature on social and personal identities. First and foremost, we have examined how consumers choose to express their social and personal identities in the context of sequential decisions. Second, we propose a key factor—expected visibility of a product involved in an initial decision—that may explain how seemingly contradictory theories of identity [31, 33, 38, 40] can ultimately coexist. Specifically, in line with identity theory and the notion of salient identity [31, 33, 38], we show that consideration of high-expected-visibility products encourages individuals to make consistent decisions that reflect their salient identities and therefore to engage in consistent behavior. On the other hand, consideration of a low-expected-visibility product might diminish the likelihood of engaging in consistent behavior. In the latter case, an individual might follow Brewer’s [40] optimal distinctiveness theory and its balancing mechanism by alternating between the identities that he or she expresses across a sequence. We have also shown self-presentation concerns, triggered by high expected visibility of the product, serve as an underlying mechanism that drives the effect. Finally, we have shown that this effect only takes place in circumstances in which the identities involved in the sequence are highly integrated. In future research, it might be interesting to further explore the underlying mechanism driving behavior when the first product is of low expected visibility, and to consider additional variables that might generate inconsistent behavior.

### 10.2 Managerial implications

Marketers face numerous scenarios in which they seek to encourage consistency in consumer behavior; for example, they might attempt to encourage repeated purchases or consistent consumption of healthy food. Alternatively, they might seek to encourage inconsistent behavior, e.g., by encouraging occasional consumption of hedonic products among individuals who generally lead a utilitarian lifestyle. These types of decisions affect the identity expression of the individual. Our findings suggest that marketers should be aware of the potential effects of the visibility of the consumed product on such behaviors, and consider these effects when developing marketing programs. In particular, and as presented in our experiments, marketers may be advised to present their products as having different levels of expected visibility depending on the behavior they wish to encourage. Notably, our findings suggest that making minor changes to a product’s description or presentation can have substantial effects on consumer choices. Thus, marketers may be able to manage levels of expected product visibility relatively easily by modifying the visual imagery or the product descriptions used in their communications. It may also be beneficial to adjust the visibility of the consumed product by managing aspects of product development and trying to impact a product’s consumption environment, e.g., by developing versions of a product that are intended for outdoor usage.

### 10.3 Limitations and additional directions for future research

In our experiments, we examined consistency between two decisions made in close proximity to each other. Future research could examine whether the effect of the expected visibility of the product involved in the first decision remains stable or diminishes over time, both in the context of social and personal identities, and in other contexts.

Future work can also study the effect for high commitment products, such as high priced or rarely purchased products. While there are similarities between high visibility and high commitment on the decision process, such as increased involvement in the decision [78], we believe that high commitment might not increase consumers’ self-presentation concerns, as predicted to take place under high expected visibility. We suggest future research to systematically compare the effects of product visibility under different levels of associated commitment and explore its carryover effect on consistent decisions.

Future research might also consider additional moderators for the effect of product visibility on behavioral consistency. For example, the strength of cultural norms (e.g., in collectivistic or individualistic cultures) may play a critical role in moderating the effect, such that strong cultural norms may lead individuals to rely less on the expected visibility of the consumed product and more on their desire to make decisions that are in line with socially-accepted behaviors.

## Supporting information

S1 Fig Integration between identities (based on Aron, Aron, and Smolan 1992). (DOCX)

S2 Fig Manipulating expected visibility of a consumed product by changing the description of the product (Experiment 4). (DOCX)

S3 Fig Second choice: Choosing to emphasize social or personal identity (Experiment 4). (DOCX)

S1 Table “Floodlight” analysis: The moderating role of identity integration (Experiment 3). Notes: The table presents the effect of identity integration on the change in the identity expression in the second decision. The first column shows the identity integration levels. The second column shows the effect of product expected visibility on the change in the identity expression in the second decision. The third column shows the standard errors. The fourth column shows the low level of the confidence interval and the fifth column shows the high level of the confidence interval. The sixth column shows the values of z and the seventh column shows their respective *p*-values. The Johnson-Neyman points are obtained at self-consciousness = 5.57.
(DOCX)

S2 Table Summary of results. (DOCX)
