# Supplementary figures and images for: Consistency in identity-related sequential decisions
Source: PLoS One. 2021 Dec 8;16(12):e0260048. doi: 10.1371/journal.pone.0260048 (PMC8654224; doi:10.1371/journal.pone.0260048)

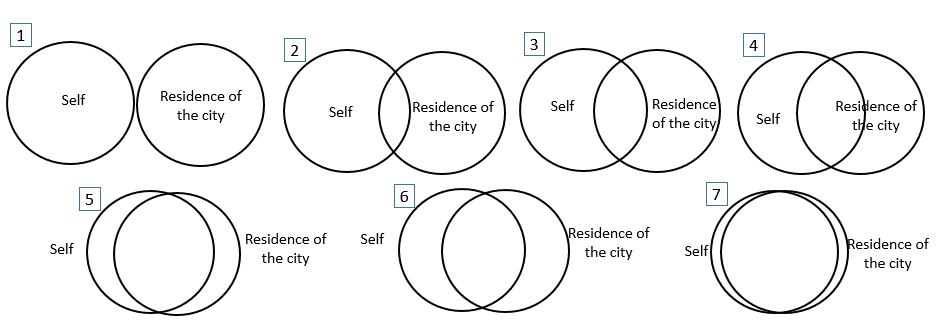

Supplement: S1 Fig — (DOCX) [file pone.0260048.s001.docx]

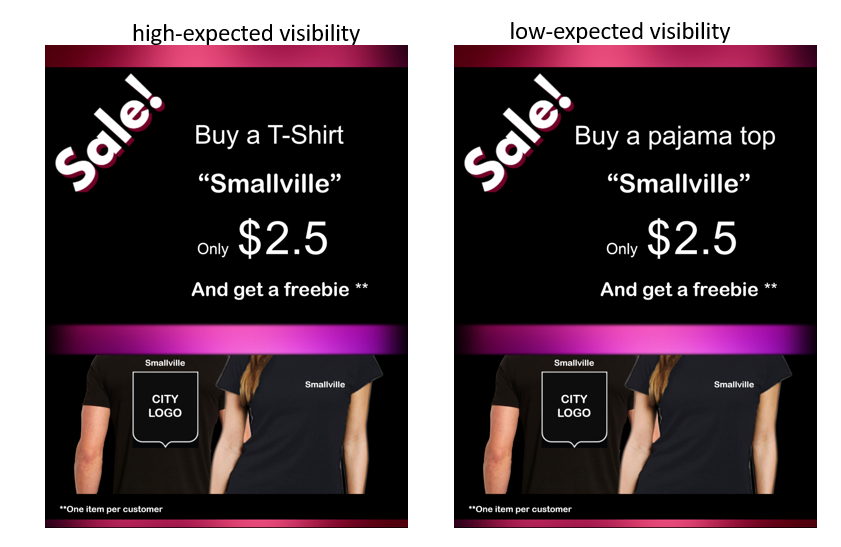

Supplement: S2 Fig — (DOCX) [file pone.0260048.s002.docx]

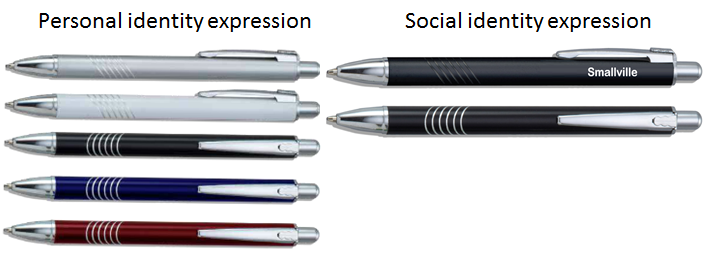

Supplement: S3 Fig — (DOCX) [file pone.0260048.s003.docx]

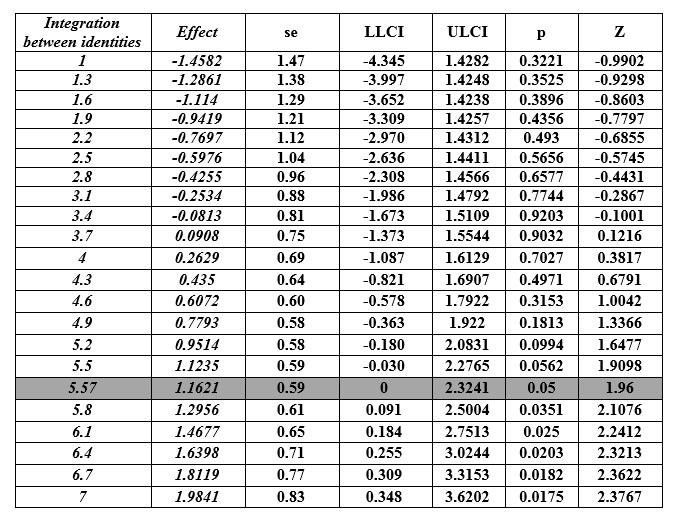

Supplement: S1 Table — Notes: The table presents the effect of identity integration on the change in the identity expression in the second decision. The first column shows the identity integration levels. The second column shows the effect of product expected visibility on the change in the identity expression in the second decision. The third column shows the standard errors. The fourth column shows the low level of the confidence interval and the fifth column shows the high level of the confidence interval. The sixth column shows the values of z and the seventh column shows their respective p-values. The Johnson-Neyman points are obtained at self-consciousness = 5.57. (DOCX) [file pone.0260048.s004.docx]
