# Supplementary material for: Consistency in identity-related sequential decisions
Source: PLoS One. 2021 Dec 8;16(12):e0260048. doi: 10.1371/journal.pone.0260048 (PMC8654224; doi:10.1371/journal.pone.0260048)
Supplement: S2 Table — (DOCX) [file pone.0260048.s005.docx]

| **Experiment** | **Identity manipulation** | **Expected visibility manipulation** | **Second choice** | **Results** |
| --- | --- | --- | --- | --- |
| Experiment 1 | Social: residence in major cities | City history meeting in a one-on-one setting (low expected visibility) vs. group setting (high expected visibility) | A t-shirt with the city logo printed on it vs. pen in a favorite color | High expected visibility: 54.0% were consistent. Low expected visibility: 38.0% were consistent.(Wald_(1)_ = 4.51, *p =.*033) |
| Experiment 2 | Personal: t-shirt in favorite color | High: t-shirt expected to be worn outdoors, so other people could see it vs.  Low: t-shirt expected to be worn at home, so that few people would see it. | Pen with the school logo printed on it vs. Pen in a favorite color  (participants were all students of the same school) | High expected visibility: 83.8% were consistent. Low expected visibility: 63.4% were consistent.  (Wald_(1)_ = 3.923, p = .048) |
| Experiment 3 | Social: Metropolis residence | 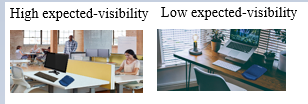 | Pen with the Metropolis logo printed on it vs. pen in a favorite color | *Moderation analysis: b*= 1.18, *SE* = 0.60, z(1) = 1.99, *p* = .047 |
| Experiment 4 | Social: Smallville residence | High: T-shirt vs. Low: Pajama top | Pen with the Smallville logo printed on it vs. pen in a favorite color | High expected visibility: 74% were consistent. Low expected visibility: 35.5% were consistent.  (Wald_(1)_ = 3.52, p = .06) |
| Experiment 5 | Personal: t-shirt in favorite color vs. social: t-shirt with the school  (participants were all students of the same school) | High: t-shirt expected to be worn outdoors, so other people could see it vs.  Low: t-shirt expected to be worn at home, so that few people would see it. | Pen with the school logo printed on it vs. pen in a favorite color | High expected visibility: 57.7% were consistent. Low expected visibility: 36.0% were consistent.  (Wald_(1)_ = 4.03, p = .030) |
